# Does exercise in cool water cause a higher risk of hypoglycaemia than in thermoneutral conditions in type 1 diabetes?

**DOI:** 10.1007/s00125-025-06456-w

**Published:** 2025-06-18

**Authors:** Kristina J. Abramoff, Shane K. Maloney, Timothy W. Jones, Elizabeth A. Davis, Paul A. Fournier

**Affiliations:** 1 Department of Sport Science, Exercise and Health, School of Human Sciences, University of Western Australia, Crawley, WA Australia; 2 Department of Anatomy, Physiology and Human Biology, School of Human Sciences, University of Western Australia, Crawley, WA Australia; 3 Children’s Diabetes Centre, The Kids Research Institute, Nedlands, WA Australia

**Keywords:** Blood glucose, Cool-water cycling, Hypoglycaemia, Thermoneutral water cycling, Type 1 diabetes mellitus, Water immersion

## Abstract

**Aims/hypothesis:**

The aim of this study was to test the hypothesis that exercise in cool water results in a greater decrease in blood glucose concentration than in thermoneutral water or on land in individuals with type 1 diabetes.

**Methods:**

Eight overnight-fasted individuals (aged 18–40 years) with type 1 diabetes completed 3 × 60 min cycling sessions on an ergometer at 40% of their on-land $\dot{V}{\text{O}}_{\text{2peak}}$ under the following conditions: while immersed in cool water (22°C) or in thermoneutral water (32°C) or on land at thermoneutrality (22°C). At time intervals, the following variables were measured: concentration of blood glucose and plasma insulin, skin blood flow, skin temperature and rate of carbohydrate and fat oxidation.

**Results:**

Blood glucose concentration did not change in response to cycling while immersed in cool or thermoneutral water (*p*>0.05) but decreased during cycling on land (*p*<0.05). The concentration of plasma insulin decreased during and early after cycling in cool water (*p*<0.05). During 60 min of on-land recovery (at 24°C) after cycling in cool water, blood glucose concentration increased significantly (~2 mmol/l, *p*<0.05), but not after cycling in thermoneutral water or on-land.

**Conclusions/interpretation:**

Exercise at 40% $\dot{V}{\text{O}}_{\text{2peak}}$ performed in a basal insulinaemic state in cool water in people with type 1 diabetes does not cause a greater decrease in blood glucose concentration than in thermoneutral water or on land, but blood glucose increases early during on-land recovery, probably as a result of a transient fall in plasma insulin.

**Graphical Abstract:**

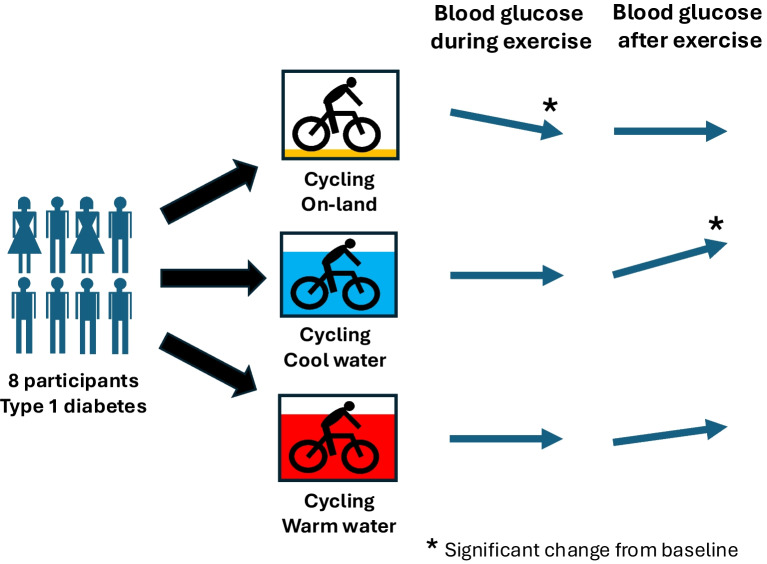


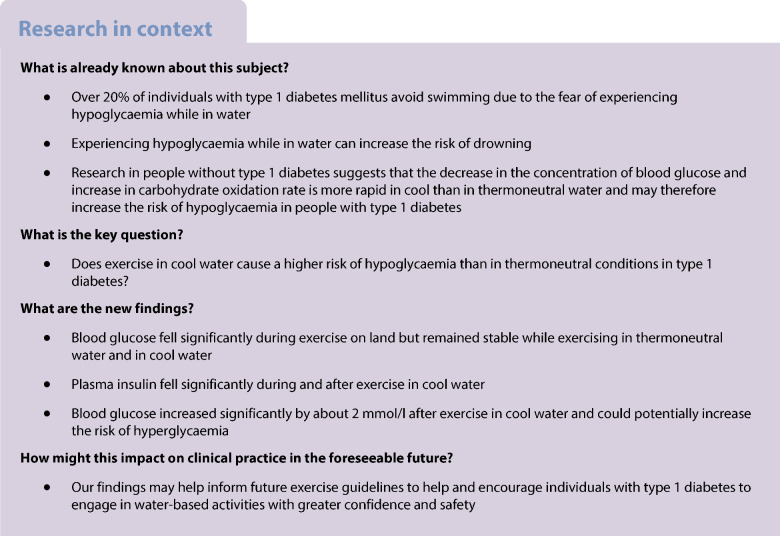


## Introduction

Current guidelines for diabetes management provide recommendations that aim to help people with type 1 diabetes mellitus adjust their insulin dose and carbohydrate intake to reduce the risk of hypoglycaemia during and after exercise [1–3]. One major limitation of these guidelines is that they generally provide little information on the impact that some environmental conditions can have on blood glucose during exercise. In particular, the effect of water temperature on blood glucose response to exercise in water is an issue that has never been examined in people with type 1 diabetes, which is regrettable considering the popularity of water-based activities.

There is indirect evidence that blood glucose concentration may not decrease as rapidly if exercise is performed in cool or cold water. Indeed, we and others have shown that both skin temperature and subcutaneous blood flow decrease in response to cold exposure [4–8], with the resulting decrease in the insulin absorption rate from its injection site under the skin leading to a lower concentration of plasma insulin in people with type 1 diabetes [4, 5, 7]. If such a decrease in plasma insulin were to occur during exercise in cool/cold water, this would be expected to oppose the glucose-lowering effect of exercise and thus decrease the risk of hypoglycaemia compared with exercise under warmer conditions. On the other hand, research in people without type 1 diabetes suggests that exercise in cool or cold water may increase the risk of hypoglycaemia in people with type 1 diabetes [9–13]. Indeed, the decrease in blood glucose concentration [9] and the increased rate of carbohydrate oxidation [9–13] in people without type 1 diabetes are more pronounced in cool than in thermoneutral water [9]. It is timely to investigate whether exercise in cool water is a risk factor for hypoglycaemia in people with type 1 diabetes.

This examination is crucial due to the widespread occurrence of cool to cold water temperatures (21–23°C) throughout the year at numerous popular beaches worldwide [14] and the popularity of beachgoing and participation in beach activities such as swimming, wading in water, playing in waves or snorkelling [15–17]. Also, more than 20% of people with type 1 diabetes abstain from swimming due to their fear of experiencing an episode of hypoglycaemia in water [18], which is understandable since hypoglycaemia while in water elevates the risk of drowning [19, 20]. Since it is unclear whether exercise in cool/cold water is a risk factor for hypoglycaemia, addressing this issue may help update future blood glucose management guidelines for people with type 1 diabetes. Therefore, the primary aim of our study was to test the hypothesis that exercise at 40% $\dot{V}{\text{O}}_{\text{2peak}}$ in individuals with type 1 diabetes results in a larger decrease in the concentration of blood glucose in cool water (22°C) compared with thermoneutral water (32°C) or on land (22°C). The findings thus obtained may help update future blood glucose management guidelines for people with type 1 diabetes.

## Methods

### Participants

The study aimed to recruit eight healthy, non-obese individuals with type 1 diabetes aged 18–40 years, with no restriction on ethnicity and no history of regular cool-water exposure or cold acclimatisation. Exclusion criteria included pregnancy, any medical condition other than type 1 diabetes and the use of automated insulin delivery devices. All female participants were tested during the follicular phase of their menstrual cycle, which required testing sessions to take place across separate cycles. Gender was self-reported, and all participants identified with their assigned sex at birth. This sample size was large enough to detect a significant difference between the treatments with a statistical power of 0.8 and an effect size of 0.9 at a significance level of *p*<0.05. Written informed consent was obtained from each participant, and ethics approval was granted by the Human Research Ethics Office at the University of Western Australia (RA-4-20-4563).

### Experimental design

All participants attended a familiarisation session followed by three exercise sessions each on different days. During the familiarisation session, the participants were familiarised with our research team, equipment and procedures, including the exercise protocol. Height and body mass were recorded as well as body composition (% fat mass) using dual-energy x-ray absorptiometry (iDXA, GE Healthcare, Madison, WI, USA). Participants with obesity were excluded from the study because excessive adiposity can affect the response of body temperature to cold exposure [21]. The participants then had their $\dot{V}{\text{O}}_{\text{2peak}}$ measured on a cycle ergometer on land as outlined below (Monark Exercise, Vansbro, Sweden).

Before each of the three exercise sessions, the participants completed an exercise/sleep/insulin diary and a food diary, and were required to eat their most common meal the day before testing, not to alter their basal insulin regimen and timing and not to consume any alcohol or caffeine [22]. The total kilojoule intake and macronutrient breakdown of their diets were calculated using the MyFitnessPal app (MyFitnessPal, 2022, USA). Each exercise session took place at the same time in the morning after an overnight fast while the participants were under the influence of only either slow acting insulin previously administered in the abdomen or rapid acting insulin infused at a basal rate, and testing was allowed only if their blood glucose concentration was between 5 and 10 mmol/l. The participants who used insulin pumps kept them connected and secured on the top of their shoulder to avoid water exposure. These insulinaemic conditions were adopted because they are associated with a low risk of hypoglycaemia during exercise [2, 3] as they result in only a small decrease in blood glucose concentration during on-land exercise in people with type 1 diabetes [23, 24].

The in-water exercise protocol required the participants to cycle on a modified FitMax Aqua Bike Pool Exercise (FitMax, USA) ergometer while being at a water depth that was level with the participants’ sternoclavicular notch. In-water cycling was adopted because: (1) it allowed the participants to remain stationary in the water, making it easier to collect data compared with swimming or wading; (2) most individuals can cycle competently in water for 1 h with little prior training as opposed to swimming, thus facilitating the recruitment of participants; and (3) the upright body position during cycling and the movement of the legs mimics the action of treading water [25].

A randomised, counterbalanced, repeated measures study design was adopted to compare the effect of exercising in cool water or thermoneutral water or on land on the blood glucose concentration during and early after exercise. All three exercise sessions lasted 60 min and were performed at an intensity of 40% of on-land $\dot{V}{\text{O}}_{\text{2peak}}$ and at a cadence of 60 rev/min. This exercise intensity was adopted because it is well tolerated and matches the exercise intensity of past studies in individuals without type 1 diabetes [10]. One exercise session was completed in cool water (COOL, 22°C), the other in thermoneutral water (THERMO, 32°C) [26] and another session took place on land at a temperature that does not require active thermogenesis to achieve heat balance at rest (LAND, 22°C) [27], with these three testing sessions taking place at least 7 days apart. The temperature of 22°C for the COOL condition was adopted because: (1) it is conducive to shivering without causing a major decrease in core temperature in response to 1 h of water immersion at rest [28]; (2) the temperature of the water at many beaches is cool (21–23°C) [14]; and (3) it is well tolerated by most participants. After each testing session, the participants were dried off and then remained for 1 h in a room at 24°C, a temperature chosen to maintain thermal balance without relying on any shivering thermogenesis [26]. This study was therefore not designed to evaluate whether our experimental conditions affected the risk of late onset hypoglycaemia. Since the participants were in a fasted basal insulinaemic state at the time of testing, our experimental design and chosen ambient temperature imitate the conditions of beachgoing that can be found before breakfast or late into the afternoon [28].

Before, and at 15 min intervals during and after exercise, the following variables were measured: capillary concentration of glucose and lactate (lactate was measured using a Lactate Pro 2 Analyzer; ARKAY, Japan) in the blood taken from a fingertip, expired gases to measure energy consumption and the rate of carbohydrate and fat oxidation, core temperature, both skin temperature and subcutaneous blood flow near the insulin administration site, heart rate and the underwater electromyography (EMG) activity of the deltoid and trapezius muscles to detect involuntary muscle contraction caused by shivering without interference from voluntary muscle contraction, such as in the leg muscles. Before, during and after exercise, venous blood was also sampled to assay the plasma concentration of free insulin. The protocols to sample blood and measure the aforementioned variables are described in detail in our previous work [5].

### Determination of $\dot{V}{\text{O}}_{\text{2peak}}$

After a 5 min warm-up at 25 W on a Monark, model 686 cycle ergometer (Monark Exercise, Vansbro, Sweden) at a constant pedal rate of 60 rev/min, the $\dot{V}{\text{O}}_{\text{2peak}}$ test started with unloaded cycling for 2 min. Thereafter, the workload was increased by 29 W every 3 min until an intensity of 118 W. Then, the workload was increased in 15 W increments [29]. The criteria for the attainment of $\dot{V}{\text{O}}_{\text{2peak}}$ included volitional exhaustion, a respiratory exchange ratio (RER) >1.1 or a plateau in $\dot{V}{\text{O}}_{2}$, operationally defined as a change in $\dot{V}{\text{O}}_{2}$ <50 ml/min with an increase in the workload [30]. Expired air was collected and analysed using a pre-calibrated indirect calorimetry system (TrueOne 2400, Parvo Medica, UT, USA).

### Statistical analyses

The area between the variable × time curve and baseline values (area between the baseline and curve [ABC]) was compared between conditions to keep the risk of type 1 error to a minimum. Our results were also analysed using a two-way repeated measures ANOVA with a Bonferroni correction for post hoc analysis. Cohen’s *d* effect sizes were also calculated using the Statistical Package for the Social Sciences (SPSS) and interpreted in accordance with previous recommendations [31]. All statistical analyses were performed using SPSS version 25.0 (IBM, New York, NY, USA). With the exception of the descriptive characteristics of the participants that are expressed as mean (SD), all of the results are expressed as mean ± SEM with significance set at *p*<0.05.

## Results

### Descriptive characteristics of the participants

Eight healthy, complication-free individuals with type 1 diabetes (two female and six male) were recruited for the study (descriptive characteristics are provided in Table 1). Our participants self-identified their ethnicity as Asian (*n*=1) or white (*n*=7).

**Table 1:** Descriptive characteristics of participants (*n*=8)

Characteristic	Mean (SD)/*N*
Age (years)	29.9 (8.4)
Height (cm)	177.0 (8.0)
Male	6
Female	2
Body mass (kg)	78.2 (13.2)
BMI (kg/m^2^)	24.7 (2.7)
Adipose tissue (%)	20.0 (11.1)
HbA_1c_ (mmol/mol)	51.8 (7.9)
HbA_1c_ (%)	6.9 (2.9)
$\dot{V}{\text{O}}_{\text{2peak}}$ (ml min^−1^ kg^−1^)	35.1 (7.0)
Duration of diabetes (years)	15.1 (7.4)
Total daily dose of insulin (units/day)	51 (10)
Total daily dose of insulin (units kg^−1^ day^−1^)	0.68 (0.21)
Treatment with MDI	6
Levemir (MDI)	1
Lantus (MDI)	5
Treatment with insulin pump	2
Novorapid (Medtronic, MiniMed 640G)	1
Humalog (Medtronic, MiniMed 670G)	1

MDI, multiple daily insulin

### Matching of experimental conditions

The ambient temperature and humidity of the recovery room did not differ between the experimental conditions (*p*>0.05; Table 2). On the day prior to testing, there was no significant difference in the intake of carbohydrate, fat, protein and total energy as well as in sleep duration and quality between the conditions (*p*>0.05; Table 3). Mean ± SEM exercise intensity did not differ (*p*>0.05) between COOL (12.07  ± 1.13 ml min^−1^ kg^−1^), THERMO (10.21 ± 1.62 ml min^−1^ kg^−1^) and LAND (11.67 ± 1.07 ml min^−1^ kg^−1^; Table 2).

**Table 2:** Mean exercise intensity and environmental conditions during the recovery period of each testing session (*n*=8)

Variable	Trial	Mean (SD)	*p* value
Mean $\dot{V}{\text{O}}_{2}$ during exercise (ml min^−1^ kg^−1^)	Cool water	12.07 (3.20)	C vs T, *p*=0.37
	Thermoneutral water	10.21 (4.58)	C vs L, *p*=0.95
	Land	11.67 (3.03)	T vs L, *p*=0.53
Air temperature (°C)	Cool water	25.0 (2.6)	C vs T, *p*=0.72
	Thermoneutral water	25.4 (2.3)	C vs L, *p*=0.34
	Land	24.2 (0.8)	T vs L, *p*=0.41
Relative humidity (%)	Cool water	42.9 (7.6)	C vs T, *p*=0.60
	Thermoneutral water	46.1 (11.9)	C vs L, *p*=0.20
	Land	46.6 (3.7)	T vs L, *p*=0.91

C, cool water; L, land; T, thermoneutral water

**Table 3:** Sleep quantity and macronutrient intake of the participants on the day prior to testing for each condition (*n*=8)

Variable	Trial	Mean (SD)	*p* value
Sleep (h)	Cool water	7.2 (1.8)	C vs T, *p*=0.84
	Thermoneutral water	7.3 (2.1)	C vs L, *p*=0.27
	Land	8.2 (0.2)	T vs L, *p*=0.43
Carbohydrate (g/day)	Cool water	210 (83)	C vs T, *p*=0.80
	Thermoneutral water	227 (52)	C vs L, *p*=0.97
	Land	205 (38)	T vs L, *p*=0.67
Fat (g/day)	Cool water	88.8 (13.5)	C vs T, *p*=0.56
	Thermoneutral water	93.3 (17.0)	C vs L, *p*=0.70
	Land	98.0 (14.4)	T vs L, *p*=0.86
Protein (g/day)	Cool water	94.3 (15.7)	C vs T, *p*=0.93
	Thermoneutral water	98.0 (13.9)	C vs L, *p*=0.99
	Land	95.5 (20.3)	T vs L, *p*=0.96
Total energy (kJ/day)	Cool water	8847 (848)	C vs T, *p*=0.99
	Thermoneutral water	8964 (639)	C vs L, *p*=1.00
	Land	9055 (985)	T vs L, *p*=0.99

C, cool water; L, land; T, thermoneutral water

### Blood glucose

**Fig. 1:**
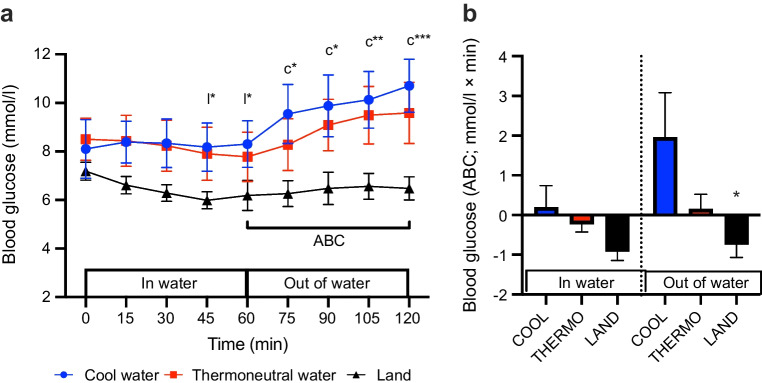
Effect of cycling in cool water, in thermoneutral water or on land on the concentration of blood glucose (mmol/l) (**a**) over time and (**b**) displayed as ABC. Data are displayed as mean ± SEM (*n*=8). *p<0.05, **p<0.01, ***p<0.001: (**a**) c, l, significantly different from pre-exercise level in COOL and LAND, respectively. (**b**) *p<0.05 significant difference in ABC compared with COOL

At 60 min of exercise, there was no significant change in blood glucose from pre-exercise level in COOL or THERMO (*p*>0.05), but blood glucose concentration was significantly lower in LAND (*p*<0.05; Fig. 1, Table 4). After 60 min of recovery, blood glucose concentration was significantly higher than it was pre-exercise in COOL (*p*<0.001), but not in THERMO or LAND (*p*>0.05; Fig. 1a, Table 4). The area between baseline and the blood glucose concentration × time curve was not different between conditions during exercise (*p*>0.05), but was significantly more positive in COOL compared with LAND during recovery (*p*<0.05; Fig. 1b, Table 5).

**Table 4:** Statistical comparisons of experimental variables between pre-exercise and 60 min of exercise (*t*=60) or 60 min of recovery (*t*=120)

Variable	Trial	*p* value (Cohen’s *d*) (*t*=0 vs *t*=60)	*p* value (Cohen’s *d*) (*t*=0 vs *t*=120)
Blood glucose (mmol/l)	Cool water	0.66	<0.001** (*d*=0.85)
	Thermoneutral water	0.12	0.13
	Land	0.03* (*d*=0.75)	0.18
Blood lactate (mmol/l)	Cool water	0.16	0.17
	Thermoneutral water	0.18	0.09
	Land	0.25	0.33
Plasma insulin (pmol/l)	Cool water	0.004** (*d*=0.75)	0.04* (*d*=0.62)
	Thermoneutral water	0.78	0.08
	Land	0.49	0.93
Core temperature (°C)	Cool water	0.14	0.003**(*d*=1.15)
	Thermoneutral water	0.12	0.85
	Land	0.04* (*d*=0.30)	0.52
Skin temperature (°C)	Cool water	0.002** (*d*=1.07)	0.75
	Thermoneutral water	0.12	0.68
	Land	0.71	0.5
Heart rate (bpm)	Cool water	0.04* (*d*=2.04)	0.66
	Thermoneutral water	0.01*(*d*=1.24)	0.25
	Land	0.0001*** (*d*=2.48)	0.13
Subcutaneous blood flow (%)	Cool water	0.03* (*d*=0.60)	0.49
	Thermoneutral water	0.62	0.02* (*d*=0.16)
	Land	0.55	0.41
EMG (deltoid) (RMS)	Cool water	0.54	0.16
	Thermoneutral water	0.94	0.79
	Land	0.89	0.73
EMG (trapezius) (RMS)	Cool water	0.14	0.34
	Thermoneutral water	0.69	0.14
	Land	0.89	0.69
Carbohydrate oxidation (g/min)	Cool water	<0.0001*** (*d*=1.55)	0.33
	Thermoneutral water	<0.0001*** (*d*=1.47)	0.51
	Land	<0.0001*** (*d*=3.57)	0.66
Fat oxidation (g/min)	Cool water	<0.0001*** (*d*=1.89)	0.15
	Thermoneutral water	<0.003*** (*d*=1.67)	0.23
	Land	<0.02* (*d*=0.57)	0.31

Statistical significance indicated as: *p<0.05, **p<0.01 and ***p<0.001

bpm, beats per min; *t*, time

**Table 5:** Statistical comparisons of area between the baseline and the variable × time curve across experimental variables

Variable	Trial	*p* value after exercise (*t*=60)	*p* value after recovery (*t*=120)
Blood glucose (mmol/l)	C vs T	0.50	0.18
	C vs L	0.08	0.04* *d*=1.16
	T vs L	0.10	0.08
Core temperature (°C)	C vs T	0.36	0.004** *d*=1.64
	C vs L	0.02* *d*=1.54	0.02* *d*=1.54
	T vs L	0.02* *d*=1.10	0.20
Skin temperature (°C)	C vs T	0.03* *d*=1.00	0.16
	C vs L	0.25	0.04 *d*=0.24
	T vs L	0.37	0.64
Subcutaneous blood flow (%)	C vs T	0.02* *d*=1.17	0.04* *d*=0.60
	C vs L	0.03* *d*=1.28	0.56
	T vs L	0.86	0.14
Carbohydrate oxidation(g/min)	C vs T	0.39	0.25
	C vs L	<0.006** *d*=0.86	0.41
	T vs L	<0.002** *d*=0.34	0.20
Fat oxidation (g/min)	C vs T	0.07	0.01* *d*=0.34
	C vs L	0.02* *d*=0.89	0.02* *d*=0.86
	T vs L	0.49	0.07

Statistical significance indicated as: *p<0.05, **p<0.01 and ***p<0.001

C, cool water; L, land; T, thermoneutral water; *t*, time

### Blood lactate

**Fig. 2:**
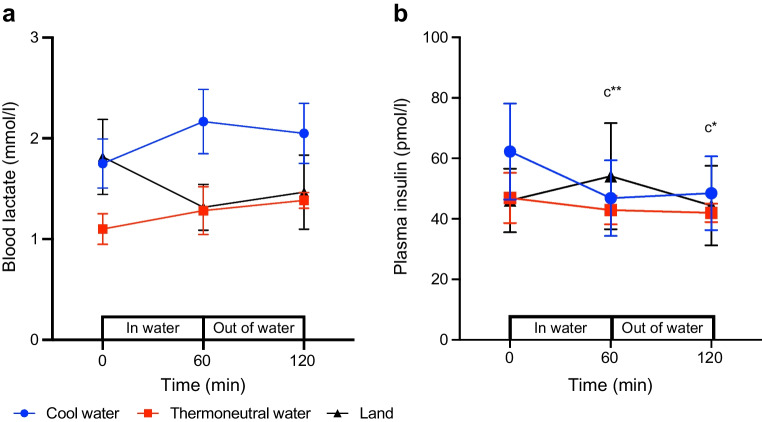
Effect of cycling in cool water, in thermoneutral water or on land on the concentration of (**a**) blood lactate (mmol/l) and (**b**) insulin. Data are displayed as mean ± SEM (*n*=8 for lactate and 6 for insulin). Of note, two of the participants experienced syncope during venepuncture, leading to the session being stopped and repeated on another day. Those participants were therefore excluded from catheterisation in all testing sessions. The analysis, based on six participants, had a statistical power of 0.75. *p<0.05, **p<0.01: c, significantly different from pre-exercise level in cool water

At 60 min of either exercise or recovery, blood lactate concentration had not changed relative to pre-exercise concentration in any of the conditions (*p*>0.05; Fig. 2a, Table 4).

### Plasma insulin

At 60 min of either exercise or recovery, there was no significant change in plasma insulin concentration relative to baseline in THERMO or LAND (*p*>0.05; Fig. 2b, Table 4). At 60 min of either exercise or recovery in COOL, plasma insulin concentration was significantly lower relative to baseline, with a moderate to large effect size (*p*<0.05; Fig. 2b, Table 4).

### Core temperature

**Fig. 3:**
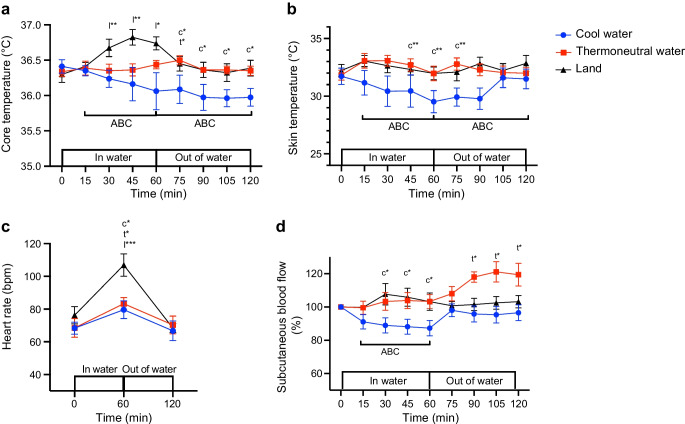
Effect of cycling in cool water, in thermoneutral water or on land on (**a**) core temperature, (**b**) skin temperature, (**c**) heart rate, and (**d**) subcutaneous blood flow expressed relative to pre-exercise level (*t*=0 min). Data are displayed as mean ± SEM (*n*=8). *p<0.05, **p<0.01, ***p<0.001: c, t, l, significantly different from pre-exercise level (*t*=0 min) in COOL, THERMO and LAND, respectively. For (**a**) core temperature: the area between the baseline and the core temperature × time curve (ABC) during exercise was significantly more positive in LAND than either COOL or THERMO. The ABC during recovery was significantly more negative in COOL than in either LAND or THERMO. For (**b**) skin temperature: the area between baseline and the skin temperature × time curve (ABC) during exercise was more negative in COOL than THERMO. The ABC during recovery was more negative in COOL than in either LAND or THERMO. For (**d**) subcutaneous blood flow: the area between baseline and the blood flow × time curve (ABC) was more negative during exercise in COOL than in either LAND or THERMO. The ABC during recovery was more positive in THERMO than in COOL. bpm, beats per min

At 60 min of exercise, core temperature did not change relative to baseline in COOL or THERMO (*p*>0.05), but had increased significantly in LAND before returning to pre-exercise level by the end of the recovery (*p*<0.05; Fig. 3a, Table 4). During recovery, core temperature did not change in THERMO (*p*>0.05), but decreased significantly in COOL (*p*<0.05; Fig. 3a, Table 4). During exercise, the area between baseline and the core temperature × time curve was more positive in LAND than in either THERMO or COOL, and was significantly more negative during recovery in COOL than in either THERMO or LAND (*p*<0.05; Fig. 3a, Table 5).

### Mean skin temperature

At 60 min of exercise, mean skin temperature had decreased relative to baseline in COOL (*p*<0.05), but it remained unchanged in THERMO or LAND (*p*>0.05; Fig. 3b, Table 4). At the end of recovery, mean skin temperature was similar to pre-exercise level in all conditions (*p*>0.05; Fig. 3b, Table 4). The area between baseline and the skin temperature × time curve was significantly more negative during exercise in COOL than in THERMO and was more negative during recovery in COOL than in LAND (*p*<0.05; Fig. 3b, Table 5).

### Heart rate

After 60 min of exercise, heart rate had increased significantly relative to baseline in all conditions, and was higher in LAND than in either COOL or THERMO (*p*<0.05; Fig. 3c, Table 4). At the end of the recovery, heart rate was back to pre-exercise level in all conditions (*p*>0.05; Fig. 3c, Table 4).

### Subcutaneous blood flow

After 60 min of exercise, subcutaneous blood flow was below pre-exercise level in COOL (*p*<0.05), but did not change in THERMO or LAND (*p*>0.05; Fig. 3d, Table 4). Subcutaneous blood flow returned to pre-exercise level within 15 min during recovery in COOL, and at 60 min of recovery it was higher in THERMO than pre-exercise level but did not change in LAND (*p*<0.05; Fig. 3d, Table 4). The area between baseline and the subcutaneous blood flow × time curve was significantly more negative during exercise in COOL than in THERMO or LAND (*p*<0.05), and was significantly more positive in THERMO than in COOL during recovery (*p*<0.05; Fig. 3d, Table 5).

### EMG amplitude of the deltoid and trapezius muscles

**Fig. 4:**
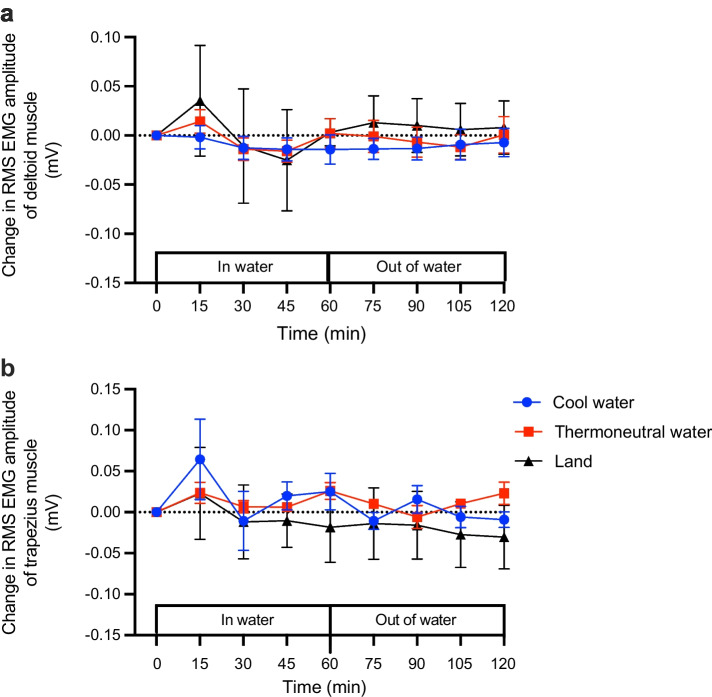
Effect of cycling in cool water, in thermoneutral water or on land on the change in RMS of EMG amplitude of the (**a**) deltoid and (**b**) trapezius muscles expressed as millivolts. EMG amplitude of the deltoid and trapezius muscles did not change during or after exercise in any of the three conditions. Data are displayed as mean ± SEM (*n*=8)

At 60 min of either exercise or recovery, there was no significant difference in root mean square (RMS) EMG amplitude in either the deltoid or trapezius muscles relative to pre-exercise amplitude in all conditions (*p*>0.05; Fig. 4a, b, Table 4).

### Rate of carbohydrate oxidation

**Fig. 5:**
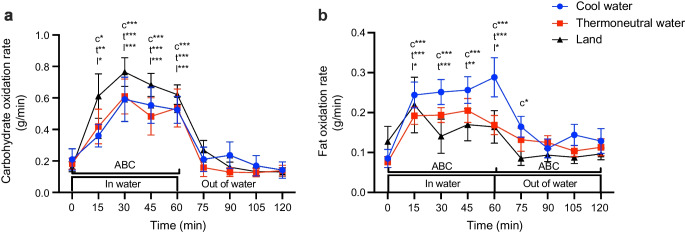
Effect of cycling in cool water, in thermoneutral water or on land on the rate of (**a**) carbohydrate and (**b**) fat oxidation. Data are displayed as mean ± SEM (*n*=8). *p<0.05, **p<0.01, ***p<0.001: c, t, l, significantly different from pre-exercise level (*t*=0 min) in COOL, THERMO and LAND, respectively. For (**a**) carbohydrate oxidation: the area between baseline and the carbohydrate oxidation rate × time curve (ABC) was more positive in LAND than in either COOL or THERMO. For (**b**) fat oxidation: the area between baseline and fat oxidation rate × time curve (ABC) was more positive in COOL vs LAND during exercise and recovery

During exercise, carbohydrate oxidation rate was significantly higher relative to pre-exercise level in all three conditions (*p*<0.0001) and was back to pre-exercise level after 60 min of recovery in all three conditions (*p*>0.05; Fig. 5a, Table 4). During exercise, the area between baseline and the carbohydrate oxidation rate × time curve was larger in LAND than in COOL or THERMO (*p*<0.05; Fig. 5a, Table 5).

### Rate of fat oxidation

During exercise, fat oxidation rate was significantly higher than pre-exercise level in all three conditions (*p*<0.05), and was back to pre-exercise level by 60 min of recovery in all three conditions (*p*>0.05; Fig. 5b, Table 4). During exercise, the area between baseline and the fat oxidation rate × time curve was more positive in COOL than in LAND, and was larger in COOL than in THERMO or LAND during recovery (*p*<0.05; Fig. 5b, Table 5).

## Discussion

We set out to test the hypothesis that physical activity at 40% $\dot{V}{\text{O}}_{\text{2peak}}$ in cool water results in a greater decrease in blood glucose concentration than in thermoneutral water or on land in individuals with type 1 diabetes. Our findings refute our hypothesis as they show that 1 h of moderate intensity exercise in cool or thermoneutral water while in a near basal insulinaemic state has no significant effect on blood glucose concentration as opposed to the decrease in blood glucose concentration that occurs during on-land exercise. Also, against expectations, early on-land recovery from exercise in cool water was associated with a ~2 mmol/l increase in blood glucose concentration, but not after thermoneutral or on-land exercise.

Our finding that exercise at 40% $\dot{V}{\text{O}}_{\text{2peak}}$ in cool or thermoneutral water had no effect on blood glucose concentration as opposed to the decrease associated with on-land exercise performed at the same metabolic intensity differs from what has been reported in people without diabetes [9, 10]. Kurobe and colleagues [10] found that blood glucose concentration in people without diabetes decreased more during swimming at 40% of $\dot{V}{\text{O}}_{\text{2peak}}$ than during on-land exercise (cycling and walking). Also, Galbo and colleagues [9] reported that, in people without type 1 diabetes, swimming at 68% of $\dot{V}{\text{O}}_{\text{2peak}}$ in water at 21°C or 27°C was associated with a more pronounced decrease in blood glucose concentration and a higher carbohydrate oxidation rate than swimming in thermoneutral water.

These different responses to exercise in cool or thermoneutral water between people with or without type 1 diabetes who exercise at 40% $\dot{V}{\text{O}}_{\text{2peak}}$ [10] might have to do with the use of different exercise protocols (underwater cycling in our study vs swimming [9]) given that different types of exercise performed at a matched intensity can affect the proportion of fat and carbohydrate that is oxidised during exercise [32]. However, it is more likely that these differences relate to the fact that our participants were treated with exogenous insulin injected subcutaneously whereas insulin in people without type 1 diabetes is of endogenous origin. Indeed, we found that cycling in cool water, but not cycling on-land or in thermoneutral water, was associated with a rapid decrease in insulin level most probably because of the cooler skin temperature and the cold-mediated decrease in subcutaneous blood flow at the site of insulin injection, an interpretation that is also supported by the work of others [5, 7]. Such a low insulin level during exercise would be expected not to favour a decrease in blood glucose concentration.

The observation that blood glucose concentration remained stable during cycling in cool or thermoneutral water but decreased slightly during on-land cycling could be explained, at least in part, by our findings that carbohydrates were oxidised at a lower rate during in-water cycling than during on-land cycling. If one assumes that the pattern of carbohydrate oxidation reflects that of the oxidation of blood glucose, blood glucose would thus be expected to have been oxidised at a lower rate during in-water cycling. Also, since exercise at 40% $\dot{V}{\text{O}}_{\text{2peak}}$ in cool water was associated with a significant decrease in the concentration of plasma insulin, this would be expected to increase hepatic glucose production in the cool-water condition as well as decrease both peripheral glucose utilisation and oxidation, thus helping to further prevent the concentration of blood glucose from decreasing during cool-water cycling.

The increase in the concentration of blood glucose during on-land recovery from exercise in cool water is consistent with earlier findings from Galbo and colleagues [9] who also reported that blood glucose concentration increased during out-of-water recovery from swimming in their non-diabetic participants. Our results are also consistent with recent findings from our laboratory [5] whereby blood glucose concentration has been reported to increase during on-land recovery from standing neck-deep in cool water in our participants with type 1 diabetes. We propose that the low rate of carbohydrate oxidation along with the maintenance of a lower than pre-exercise level of plasma insulin during on-land recovery from exercise in cool water may have contributed to the increase in glucose concentration after cycling in cool water. Also, since hypothermia is known to be associated with a decrease in insulin sensitivity [33], this would be expected to increase further hepatic glucose production in the cool-water condition as well as lead to a decrease in both peripheral glucose utilisation and oxidation rates, thus maybe contributing further to increasing blood glucose concentration.

Please note that our results also show that heart rate cannot be used instead of oxygen consumption rate to compare on-land cycling with in-water cycling. Indeed, although the metabolic intensity ($\dot{V}{\text{O}}_{2}$) was well matched between our three exercise conditions, heart rate was higher during on-land exercise than during in-water cycling. This type of finding has also been reported in people without diabetes [34] and has been explained on the basis that the hydrostatic pressure associated with water immersion causes an increase in central volume, cardiac preload and preload-mediated stroke volume, thus resulting in a lower heart rate [34]. On practical grounds, our findings imply that any study aimed at comparing on-land with in-water cycling should not rely on heart rate to match exercise intensity because doing so would result in a higher metabolic cost of exercising in water compared with on land.

The results of our study are relevant to most beachgoers as our experimental conditions (warm or cool water as well as basal insulinaemia) mimic those experienced by summer beachgoers pre-breakfast or late in the afternoon, times during daytime when the risk of both hypoglycaemia and exercise-mediated hypoglycaemia are at their lowest in people with type 1 diabetes [2, 3]. Also, the cool-water condition adopted here (22°C) falls within the range of the water temperature of many beaches globally (e.g. Australia, Brazil, California, Mexico and Spain) [14]. Although many individuals who go to the beach engage in various types of physical activities, a large proportion engage in activities such as treading water or wading, making our findings also relevant to these individuals [15, 16].

This study has some limitations. For instance, although our sample size was large enough to examine the response of our primary outcome variable, blood glucose concentration, to exercise under different conditions, the multiple comparisons performed for all our outcome variables and our small sample size have the potential to increase the risk of type II error. However, this limitation was addressed by comparing, between conditions, the area under the variable × time curve for each variable along with incorporating a Bonferroni correction for post hoc analysis. Also, the possibility of a type II error is highly unlikely for many of the comparisons reported here given their large Cohen’s *d* effect sizes and very low *p* values. It remains to be determined whether similar findings would have been obtained at higher exercise intensities or with different exercise modalities, such as swimming, meaning that one should abstain from using our findings to support any hasty generalisation/extrapolation with respect to other activity types and exercise intensities. No sex/gender analysis was conducted due to sample size being too small to perform meaningful sex/gender comparisons. Future studies with larger, more balanced sample sizes are needed to explore sex-specific differences. Another limitation is that it is unclear whether similar findings would have arisen at lower temperatures that trigger more intense shivering. Unfortunately, the use of much colder water was not an option because the high level of discomfort associated with exercise in cold water would have made the recruitment of participants even more challenging than it already was for the current study. Also, the relevance of investigating the response of blood glucose to much colder water temperatures could be questioned because people who expose themselves to such conditions typically do so while wearing a wet or dry suit.

In conclusion, on clinical grounds, our findings suggest that the risk of hypoglycaemia during and early after exercise at 40% $\dot{V}{\text{O}}_{\text{2peak}}$ while in a near basal insulinaemic state is not higher when exercise is performed in cool-water conditions that mimic those typical of many beaches compared with exercise in thermoneutral water or on land in people with type 1 diabetes. Given that many individuals with type 1 diabetes avoid water-based activities due to their fear of hypoglycaemia [18], our findings may help inform future exercise guidelines to help and encourage these individuals with type 1 diabetes to engage in water-based activities with greater confidence and safety.

## Data Availability

Some or all of the datasets generated during and/or analysed during the current study are not publicly available but are available from the corresponding author on reasonable request.

## Abbreviations

ABC: Area between the baseline and curve
COOL: Exercise session completed in cool water (22°C)
EMG: Electromyography
LAND: Exercise session completed on land (22°C)
RMS: Root mean square
SPSS: Statistical Package for the Social Sciences
THERMO: Exercise session completed in thermoneutral water (32°C)
